# Loss of long-term depression in the insular cortex after tail amputation in adult mice

**DOI:** 10.1186/1744-8069-10-1

**Published:** 2014-01-08

**Authors:** Ming-Gang Liu, Min Zhuo

**Affiliations:** 1 Center for Neuron and Disease, Frontier Institute of Science and Technology, Xi’an Jiaotong University, Xi’an 710049, China; 2 Department of Physiology, Faculty of Medicine, University of Toronto, The center for the Study of Pain, 1 King’s College Circle, Toronto, Ontario M5S 1A8, Canada

## Abstract

The insular cortex (IC) is an important forebrain structure involved in pain perception and taste memory formation. Using a 64-channel multi-electrode array system, we recently identified and characterized two major forms of synaptic plasticity in the adult mouse IC: long-term potentiation (LTP) and long-term depression (LTD). In this study, we investigate injury-related metaplastic changes in insular synaptic plasticity after distal tail amputation. We found that tail amputation in adult mice produced a selective loss of low frequency stimulation-induced LTD in the IC, without affecting (RS)-3,5-dihydroxyphenylglycine (DHPG)-evoked LTD. The impaired insular LTD could be pharmacologically rescued by priming the IC slices with a lower dose of DHPG application, a form of metaplasticity which involves activation of protein kinase C but not protein kinase A or calcium/calmodulin-dependent protein kinase II. These findings provide important insights into the synaptic mechanisms of cortical changes after peripheral amputation and suggest that restoration of insular LTD may represent a novel therapeutic strategy against the synaptic dysfunctions underlying the pathophysiology of phantom pain.

## Background

Insular cortex (IC) is an integrating forebrain structure involved in several sensory and cognitive functions, such as interoceptive awareness, taste memory, and pain perception [1-3]. In particular, human brain imaging studies have demonstrated the activation of IC in a broad range of pain conditions [4-6]. Moreover, electrical stimulation of IC directly elicits painful sensations in human subjects [7-9]. The involvement of IC in chronic pain has also been confirmed by animal experiments, showing the presence of nociceptive neurons [10,11] and pain-evoked biochemical changes [12,13] in this area. Genetic [14,15] or pharmacological [16-19] manipulation of the IC could alter the pain sensitivity. Importantly, long-term potentiation (LTP) has been revealed in the IC by both *in vivo*[20,21] and *in vitro*[22,23] electrophysiological recordings. Furthermore, neuropathic pain experience could occlude the electrical induction of insular LTP in adult mice [18], suggesting that chronic pain may share common mechanisms with insular synaptic plasticity [24].

Phantom pain refers to the feeling of pain in a body part that has been amputated [25-27]. Mechanistically, limb amputation has been shown to cause dramatic cortical reorganization in humans and primates [28-31], the amount of which correlates well with the extent of phantom pain in some reports [32-34]. We previously demonstrated that digit amputation in rats or tail amputation in mice triggered long-lasting plastic alterations in the anterior cingulate cortex (ACC), including an enhancement of excitatory synaptic responses *in vivo*[35,36], loss of long-term depression (LTD) *in vitro*[37,38] and activation of activity-dependent immediate early genes [37,39]. In addition to ACC, human imaging studies also revealed a correlation between the IC activation and phantom pain [25,40,41]. Thus, it is important to investigate the possible changes in synaptic plasticity in the IC after amputation.

It is believed that peripheral injury elicits long-lasting plastic changes in the brain via at least two major mechanisms: one is direct enhancement of excitatory synaptic transmission, and the other is loss of the ability to undergo LTD [42], also see Table 1]. In the present study, we used a 64-channel multi-electrode dish (MED64) recording system [23,38,43] to examine injury-related metaplastic changes in insular LTD caused by tail amputation in the adult mice. Our previous results demonstrated the co-existence of two different forms of LTD in the IC: NMDA receptor-dependent LTD and NMDA receptor-independent LTD [44]. Here, we report that tail amputation produces a selective loss of low frequency stimulation (LFS)-induced LTD in the adult mice IC, leaving (RS)-3,5-dihydroxyphenylglycine (DHPG)-evoked LTD intact. The impaired insular LTD could be pharmacologically rescued by priming the IC slices with application of a low-dose group I metabotropic glutamate receptor (mGluR) agonist DHPG, a form of metaplasticity that involves activation of protein kinase C (PKC) but not protein kinase A (PKA) or calcium/calmodulin-dependent protein kinase II (CaMKII).

**Table 1:** Summary of previous studies on injury-evoked changes in the induction of LTD

**Pain model**	**Species**	**Method**	**Brain region**	**Induction protocol**	**Key findings**	**Reference**
***Tail amputation***	Mouse	Field potential recording	CA1	LFS (1 Hz, 15 min)	No effect on LTD induction	[45]
				LFS (5 Hz, 3 min)		
***Partial sciatic nerve ligation***	Mouse	Field potential recording	CA1	LFS (1 Hz, 15 min)	No effect on LTD induction	[46]
***Digit amputation***	Rat	Field potential recording	ACC and parietal cortex	LFS (1 Hz, 15 min)	Lost LTD in the ACC but not parietal cortex	[37]
				LFS (5 Hz, 3 min)		
***Tail amputation***	Mouse	64-channel field potential recording	ACC	LFS (1 Hz, 15 min)	Rescue of lost LTD by mGluR1 activation	[38]
***Bone cancer pain***	Mouse	Whole-cell patch-clamp recording	ACC	Paired training	Impaired LTD accompanied by reduced NMDA receptor expression	[47]
***Tail amputation***	Mouse	64-channel field potential recording	IC	LFS (1 Hz, 15 min)	Impaired electrical LTD	The present study
				DHPG-LTD	Intact chemical LTD	

Notes: ACC, anterior cingulate cortex; DHPG, (RS)-3,5-dihydroxyphenylglycine; IC, insular cortex; LFS, low-frequency stimulation; LTD, long-term depression.

## Results

### Loss of LFS-evoked LTD in the IC after tail amputation

**Figure 1:**
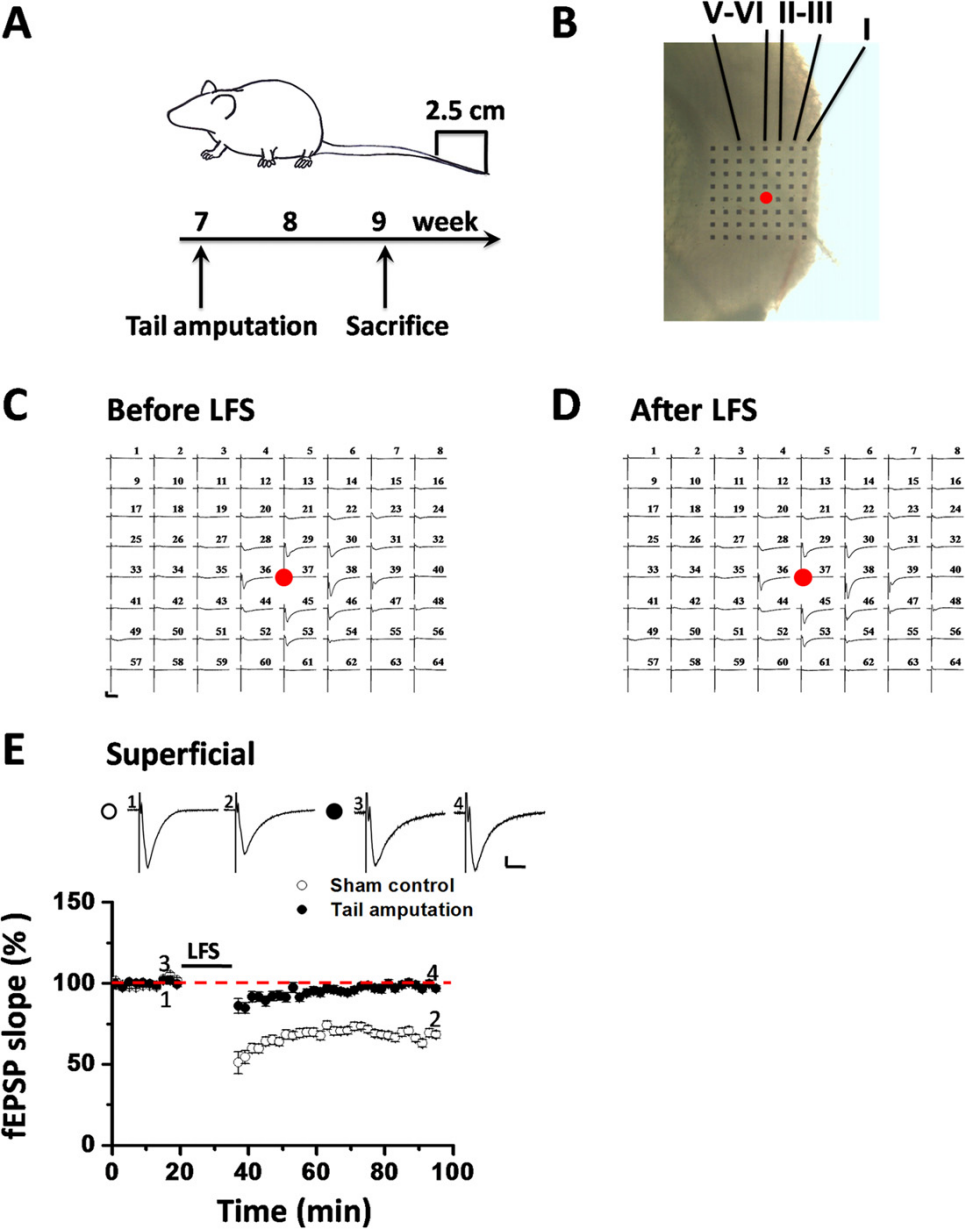
**Loss of LFS-evoked LTD in the superficial layer of the IC after tail amputation. (A)** A schematic diagram showing the tail amputation model (up) and the experimental procedure (lower). All insular slices are obtained at 2 weeks after amputation in the present study. **(B)** Light microscopy photograph showing the relative location of the IC slice with the MED64 probe, the stimulation site (red dot) and the layer designation. **(C and D)** An overview of the 64-channel multi-electrode array recordings in the tail-amputated IC slice (**C**: before LFS; **D**: 60 min after LFS). No synaptic depression was revealed. Red dots mark the stimulation sites in the deep layer. Calibration: 100 μV, 10 ms. **(E)** Pooled data of LFS-elicited LTD in the superficial layer of the IC for sham control (n = 9 slices/7 mice) and tail amputation (n = 9 slices/5 mice) groups. The sham group showed typical LTD lasting for 1 h, while tail amputation abolished the LTD induction. Sample fEPSP recordings taken at the times indicated by the corresponding numbers are shown above the plot. Calibration: 100 μV, 10 ms. Horizontal bars denote the period of LFS delivery. Error bars represent SEM.

Our previous work has demonstrated that digit or tail amputation in rats or mice could abolish the induction of LFS-evoked LTD in the ACC [37,38]. Here, we employed a previously-established 64-channel multi-electrode array system, i.e. the MED64 system [23,38,44], to examine whether the insular synaptic plasticity is equally sensitive to the amputation-induced peripheral injury in adult mice. MED64 recordings were performed in the IC slices obtained from sham control or tail-amputated mice at 2 weeks after surgery (Figure 1A). The relative location of the MED64 probe within the IC slice is shown in Figure 1B. We focused our recording sites on the rostral IC region at the level of the corpus callosum connection, where the stimulation site is usually located in the deep layer (layer V-VI) of the IC slice (the red dot in Figure 1B). One representative example recording is illustrated in Figure 1C (before LFS) and Figure 1D (60 min after LFS) for the tail-amputated group. It is clearly discerned that LFS failed to induce any depression of field excitatory postsynaptic potential (fEPSP) in this slice. The averaged data showed a complete loss of LFS-evoked LTD in the superficial layer (96.8 ± 1.9% of baseline at 60 min after LFS, n = 9 slices/5 mice, *P* = 0.701, Student’s t-test, Figure 1E). In contrast, the sham control group exhibited clear LTD of multisite synaptic responses (66.2 ± 2.5% of baseline, n = 9 slices/7 mice, *P* < 0.001, Student’s t-test, Figure 1E), which is consistent with our previous publication [44].

**Figure 2:**
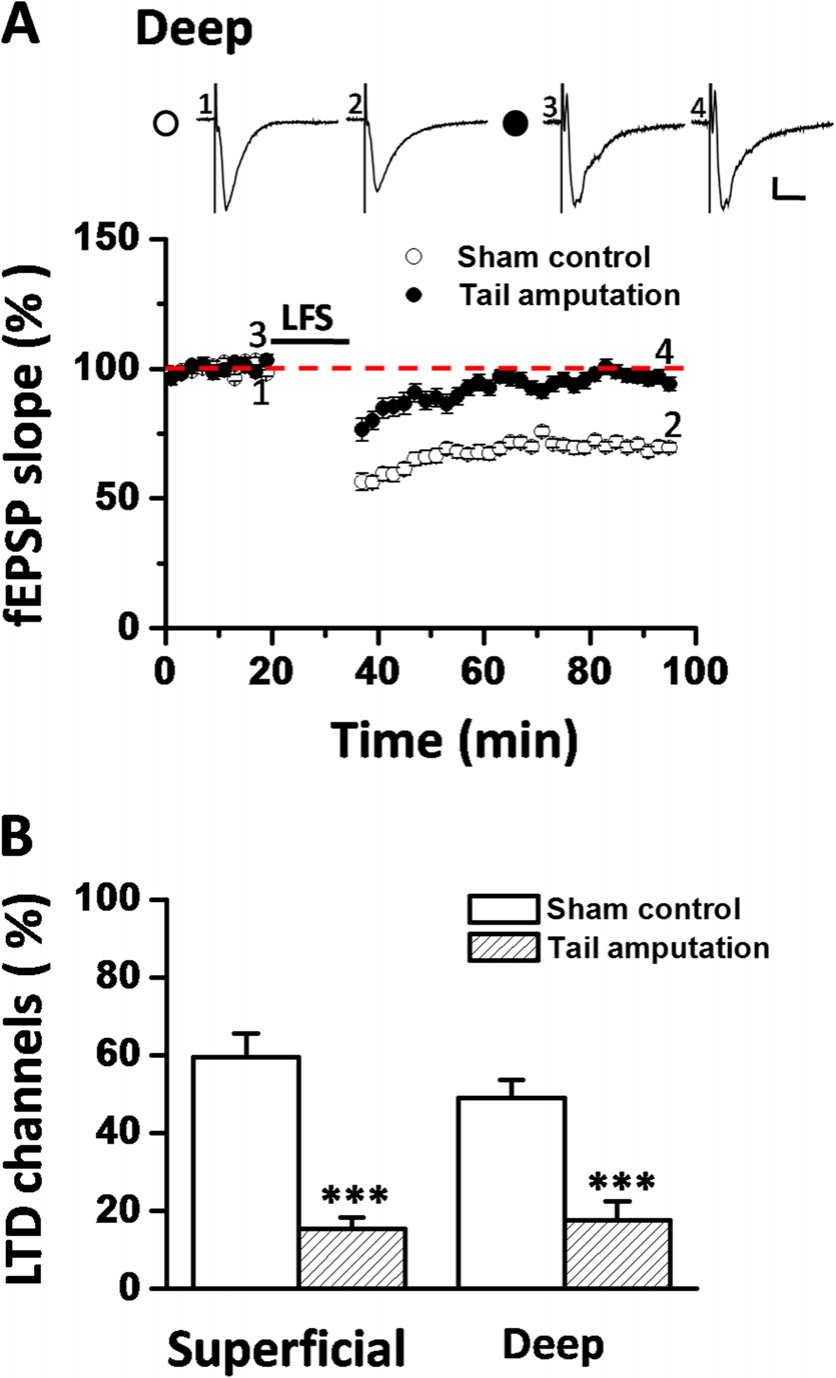
**Loss of LFS-evoked LTD in the deep layer of the IC after tail amputation. (A)** Pooled data of LFS-elicited LTD in the deep layer of the IC for sham control (n = 9 slices/7 mice) and tail amputation (n = 9 slices/5 mice) groups. Tail amputation also abolished the LTD induction in the deep layer. Sample fEPSP recordings taken at the times indicated by the corresponding numbers are shown above the plot. Calibration: 100 μV, 10 ms. Horizontal bars denote the period of LFS delivery. **(B)** Bar histogram showing the induction ratio of LTD (the percentage of LTD-showing channels among all activated channels) in the superficial layer and deep layer of the IC for sham control (n = 9 slices/7 mice) and tail-amputated (n = 9 slices/5 mice) groups. ***P < 0.001. Error bars represent SEM.

Neurons in different layers of the IC are considered to have different afferent and efferent connections with other areas of the brain, and thus may mediate distinct functions [48-50]. Therefore, we next asked whether tail amputation could also affect the LTD induction in the deep layer as it did in the superficial layer. As previously described [44], LFS application produced a long-lasting synaptic depression of fEPSPs recorded in the deep layer of the IC from the sham control group (70.3 ± 1.5% of baseline at 60 min after LFS, n = 9 slices/7mice, *P* < 0.001, Student’s t-test, Figure 2A). However, LFS failed to induce any LTD in the tail-amputated group (94.0 ± 2.6% of baseline, n = 9 slices/5 mice, *P* = 0.137, Student’s t-test, Figure 2A). Statistical analysis revealed a strong significant difference between sham and tail-amputated groups in the degree of LTD in both superficial layer (*P* < 0.001, Student’s t-test, Figure 1E) and deep layer (*P* < 0.001, Student’s t-test, Figure 2A).

Furthermore, we analyzed the induction ratio of insular LTD, defined as the percentage of LTD-showing channels among all activated channels. This ratio was greatly decreased in the tail-amputated slices (superficial layer: 15.5 ± 2.9%, *P* < 0.001, Student’s t-test; deep layer: 17.7 ± 4.8%, *P* < 0.001, Student’s t-test) compared to the sham control (superficial layer: 59.4 ± 6.0%; deep layer: 48.9 ± 4.7%, Figure 2B). Similar results were obtained with stimulation at another frequency (5 Hz, 3 min; data not shown). These findings suggest that tail amputation results in an inability of insular synapses to undergo LTD, regardless of the specific layer.

### Lack of the effect of amputation on DHPG-induced insular LTD

**Figure 3:**
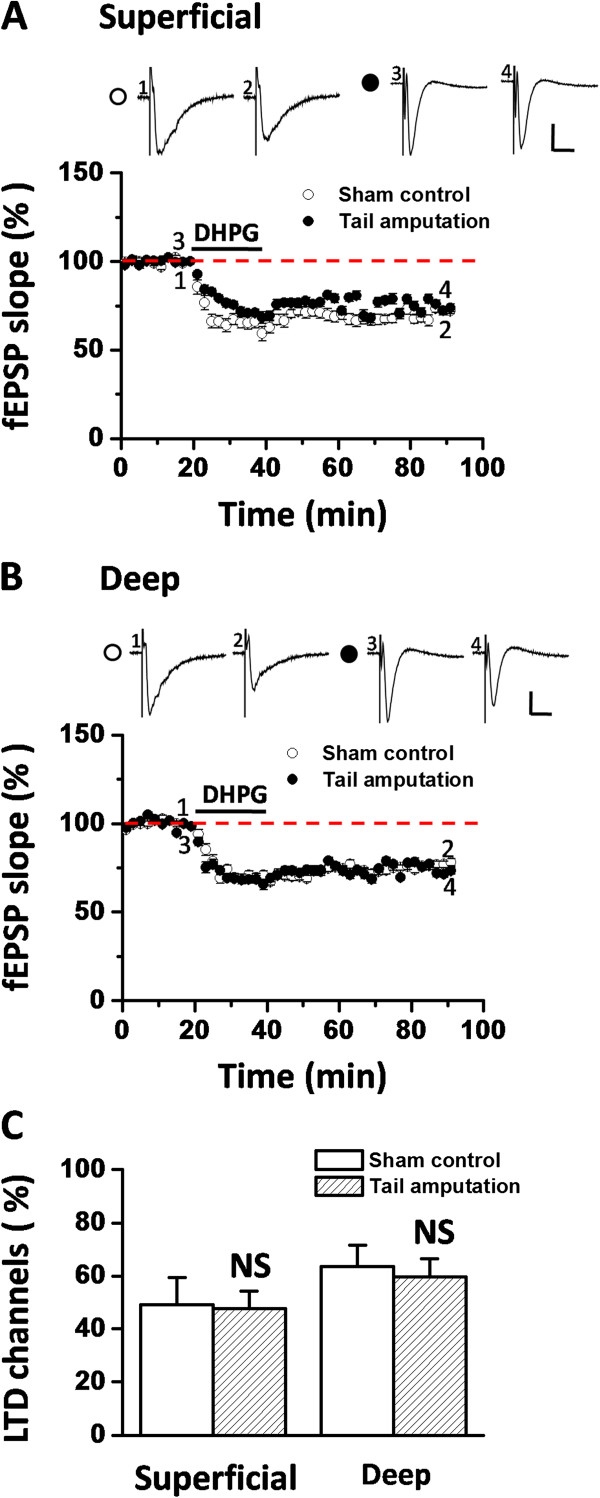
**Tail amputation has no effect on DHPG-LTD in the IC. (A and B)** Pooled data showing the comparable magnitude and temporal progression of DHPG-LTD between sham control (superficial layer: n = 7 slices/7 mice, A; deep layer: n = 8 slices/8 mice, B) and tail amputation (superficial layer: n = 9 slices/9 mice, A; deep layer: n = 9 slices/9 mice, B) groups. Sample fEPSP recordings taken at the times indicated by the corresponding numbers are shown above the plot. Calibration: 100 μV, 10 ms. Horizontal bars denote the period of DHPG application. **(C)** The induction ratio of DHPG-LTD in either superficial layer or deep layer does not differ between the two groups (sham control: n = 8 slices/8 mice; tail amputation: n = 9 slices/9 mice). NS, no significance. Error bars represent SEM.

Recently, we reported the co-existence of two distinct forms of LTD in the insular synapses: NMDA receptor-dependent LTD induced by LFS, and NMDA receptor-independent LTD induced by DHPG application [44]. Next, we sought to examine whether tail amputation could also affect the induction of DHPG-LTD. We induced DHPG-LTD by bath application of 100 μM DHPG for 20 min and then washed it out to monitor the course of chemically-induced LTD for 50 min. Similar to the previous study, DHPG infusion produced a rapid and long-lasting depression of fEPSP in the IC slices (Figure 3A and B). The synaptic responses of the superficial layer were reduced to 72.5 ± 1.8% of baseline (n = 7 slices/7 mice, *P* < 0.001, Student’s t-test, Figure 3A) at 50 min after washout of DHPG in the sham group. Interestingly, we did not observe any abolition of DHPG-LTD in the IC after tail amputation (73.6 ± 2.1% of baseline, n = 9 slices/9 mice, *P* < 0.001, Student’s t-test, Figure 3A). Similarly, the lack of effect of amputation on DHPG-LTD is also replicated in the deep layer of the IC (sham control: 77.1 ± 3.0% of baseline, n = 8 slices/8 mice, *P* < 0.001, Student’s t-test; tail amputation: 73.3 ± 2.0% of baseline, n = 9 slices/9 mice, *P* < 0.001, Student’s t-test, Figure 3B). The magnitude and duration of DHPG-LTD in the tail-amputated group did not differ from the sham control (superficial layer: *P* = 0.311, Student’s t-test, Figure 3A; deep layer, *P* = 0.303, Student’s t-test, Figure 3B). Likewise, the induction ratio of DHPG-LTD in the IC was not different between the two groups in either superficial layer (sham vs. tail amputation: 49.2 ± 10.1% vs. 47.6 ± 6.7%, *P* = 0.431, Student’s t-test) or deep layer (sham vs. tail amputation: 63.6 ± 8.1% vs. 59.6 ± 6.9%, *P =* 0.711, Student’s t-test, Figure 3C). Taken together, these results suggest that tail amputation selectively blocked the induction of LFS-evoked insular LTD, with the DHPG-LTD being intact. This result in the IC is in contrast to that in the ACC, where tail amputation prevented the occurrence of both LFS-induced LTD and mGluR1-mediated LTD [38].

### Enhanced synaptic transmission in the IC after tail amputation

**Figure 4:**
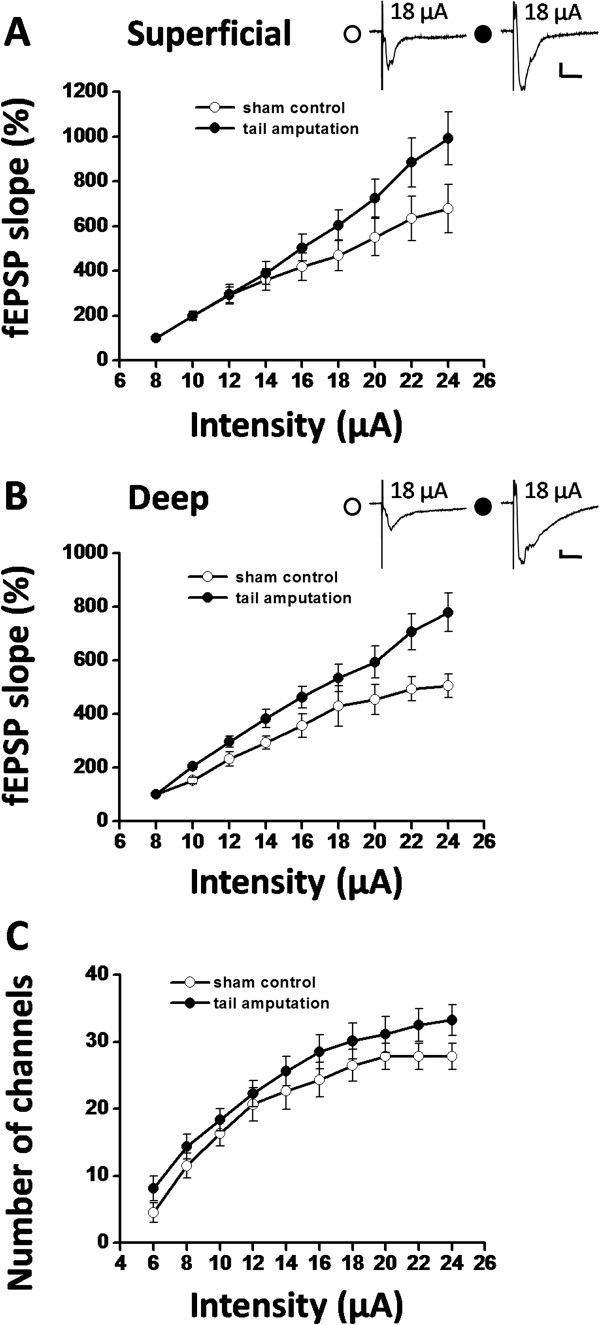
**Enhancement of synaptic transmission in the IC after tail amputation. (A)** The input–output relationship of the fEPSP slope in the superficial layer of the IC. Shown are the percentage changes of the fEPSP slope (normalized to the slope value at 8 μA) in response to series of ascending stimulation intensities. Tail amputation (n = 6 slices/4 mice) caused a leftward shift of the input–output curve compared to the sham control group (n = 6 slices/6 mice). **(B)** Pooled data of the input–output relationship of the fEPSP slope in the deep layer of the IC. Similarly, tail amputation (n = 6 slices/4 mice) resulted in a leftward shift of the curve compared to the sham control (n = 5 slices/5 mice). The insets in (A) and (B) show the representative fEPSP traces recorded at 18 μA for both sham (left) and tail-amputated (right) groups. Calibration: 100 μV, 10 ms. **(C)** The input-out curve of the number of activated channels obtained at graded stimulation intensities in the IC slice. Significant difference was detected between the sham control (n = 6 slices/6 mice) and tail-amputated (n = 8 slices/5 mice) group at higher stimulation intensities. Error bars represent SEM.

It has been previously reported that peripheral inflammation or nerve injury could trigger a long-term enhancement of excitatory synaptic transmission in various brain regions, such as ACC [51-54], amygdala [55-57], and hippocampus [58]. We next examined whether similar alterations in synaptic efficacy could be elicited in the IC after peripheral injury. The input–output relationships, measuring fEPSP slope (output) as a function of the afferent stimulus intensity (input), were compared between sham control and tail-amputated (two weeks) groups. The slope of the curve was evidently shifted to the left at higher stimulation intensities after amputation (n = 6 slices/4 mice for both superficial layer and deep layer), compared with that in control group (n = 6 slices/6 mice for superficial layer; n = 5 slices/5 mice for deep layer) (Figure 4A and B). These results suggest that excitatory synaptic transmission is likely enhanced following tail amputation experience. Nevertheless, the curves did not move leftward in a parallel manner, indicating no alteration in the threshold for inducing fEPSPs. Furthermore, the input–output curves of the total number of activated channels showed a similar trend between the two groups (n = 6 slices/6 mice for sham control; n = 8 slices/5 mice for tail-amputated group; Figure 4C).

### Pharmacological rescue of LFS-evoked insular LTD after tail amputation

**Figure 5:**
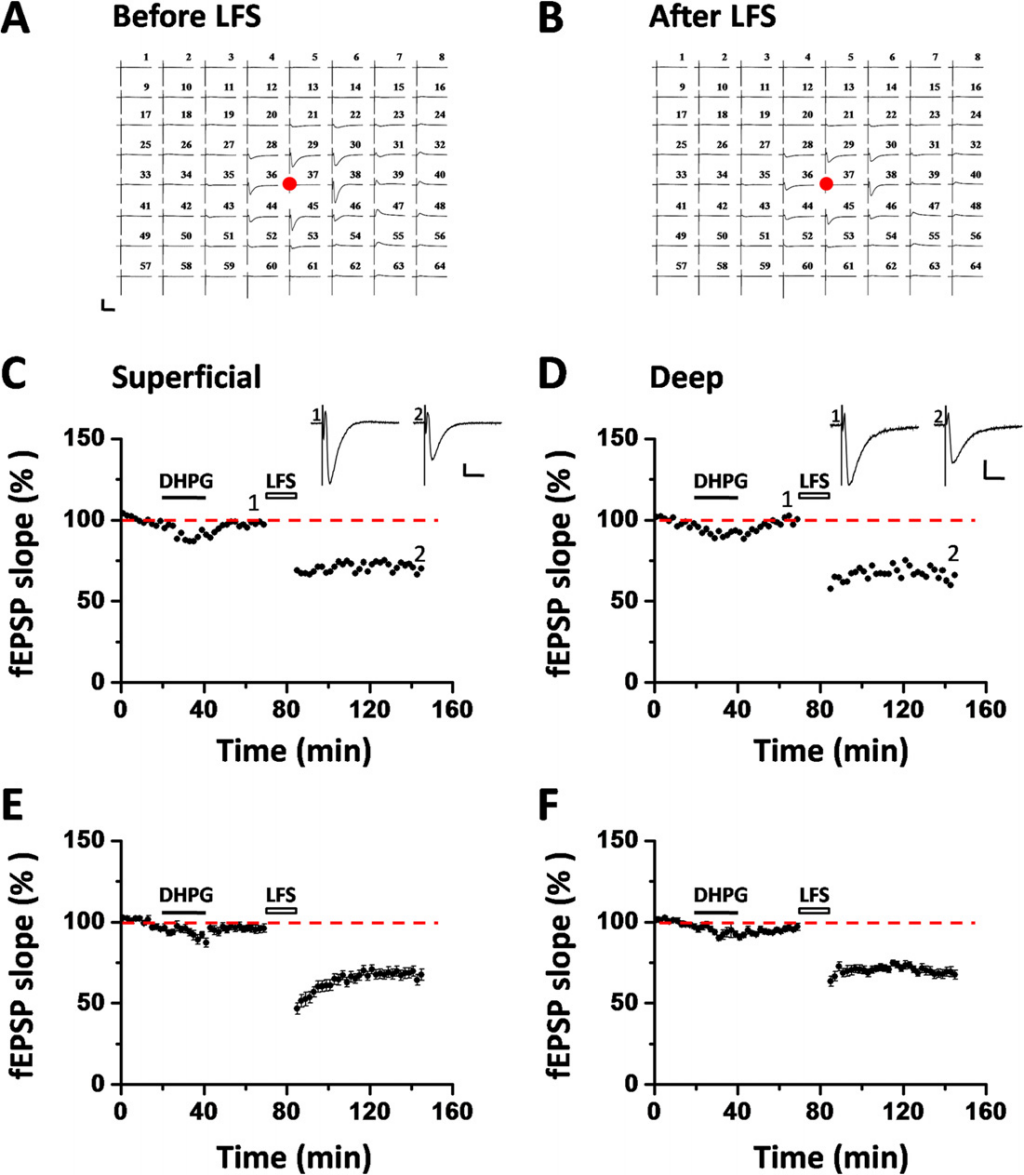
**Pharmacological rescue of LFS-evoked insular LTD in the tail-amputated mice by group I mGluR activation. (A and B)** An overview of 64-channel multi-electrode array recordings in the tail-amputated IC slice (**A**: before LFS; **B**: 60 min after LFS). DHPG (20 μM) was applied for 20 min followed by washout for 30 min. Then LFS was given. Bath application of low dose of DHPG only elicited acute depression. However, subsequent LFS induced clear LTD after priming with DHPG. Red dots denote the stimulation sites in the deep layer. Calibration: 100 μV, 10 ms. **(C and D)** One representative example slice showing the rescue of LFS-induced LTD by DHPG priming in both superficial layer **(C)** and deep layer **(D)** of the IC. Inset traces show representative fEPSPs at the time points indicated by the numbers in the graph. Calibration: 100 μV, 10 ms. **(E and F)** Summarized data for the superficial layer (n = 6 slices/5 mice) and deep layer (n = 5 slices/5 mice). Horizontal bars denote the period of DHPG application or LFS delivery as indicated. Error bars represent SEM.

Prior activation of group I mGluRs could produce metaplastic effects on synaptic plasticity in the hippocampus, shown as a large enhancement in the induction of hippocampal LTP [59,60], for review, see [61]. Our previous work revealed another form of group I mGluR-mediated metaplasticity in the ACC, that is, priming ACC slices with pharmacological activation of mGluR1 rescued the loss of LTD caused by the tail amputation [38]. Here, using the same rationale, we attempted to rescue LFS-induced insular LTD by priming the IC slices with bath application of a lower dose of DHPG (20 μM, 20 min). Figure 5A and B illustrates the overview of the 64-channel recordings obtained before LFS and 60 min after LFS in one DHPG-primed and tail-amputated IC slice. DHPG treatment at this dose failed to trigger any LTD of multisite synaptic responses, but only a rapid and transient acute depression was observed in either superficial layer (90.8% of baseline at the end of DHPG infusion, Figure 5C) or deep layer (92.4% of baseline at the end of DHPG infusion, Figure 5D). However, subsequent LFS indeed led to a significant depression of the fEPSPs in a single example (superficial layer: 70.0% of baseline at 60 min after LFS, Figure 5C; deep layer: 65.9% of baseline at 60 min after LFS, Figure 5D) and in pooled data (superficial layer: 67.5 ± 3.5% of baseline, n = 6 slices/5 mice, *P* = 0.002, Student’s t-test, Figure 5E; deep layer: 67.5 ± 2.9% of baseline, n = 5 slices/5 mice, *P* = 0.008, Student’s t-test, Figure 5F). The magnitude of DHPG-rescued LTD in the tail-amputated mice is similar to that of the sham control mice (compare Figure 5E and F with Figure 1E and Figure 2A). These results indicate that similar to the ACC synapses, prior activation of group I mGluRs can produce a form of metaplasticity that restores the LFS-evoked LTD in the IC in the tail-amputated mice.

### Protein kinase C, but not CaMKII or PKA, is involved in the rescue of insular LTD

**Figure 6:**
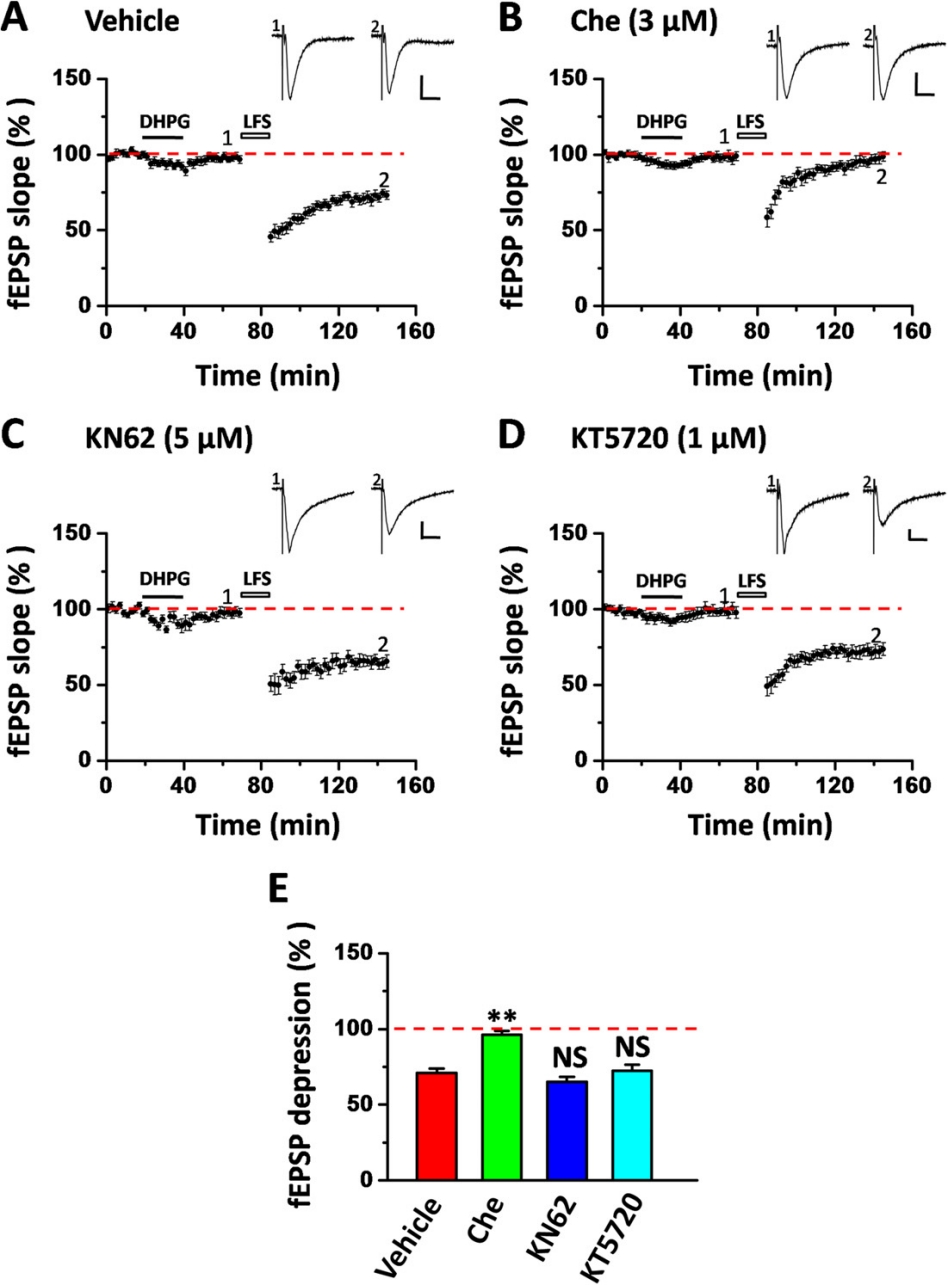
**PKC, but not CaMKII or PKA, is involved in the rescue of LFS-evoked insular LTD in the superficial layer. (A)** Vehicle control group exhibited the normal rescue of insular LTD (n = 5 slices/3 mice). **(B)** Co-application of the PKC inhibitor chelerythrine (Che, 3 μM) together with DHPG (20 μM, 20 min) blocked the LTD rescue (n = 6 slices/6 mice). **(C)** CaMKII inhibitor KN62 (5 μM) could not affect the LTD recovery (n = 6 slices/5 mice). **(D)** PKA inhibitor KT5720 (1 μM) had no effect on DHPG-primed insular LTD (n = 7 slices/5 mice). Inset traces in **(A-D)** show representative fEPSPs at the time points indicated by the numbers in the graph. Calibration: 100 μV, 10 ms. Horizontal bars denote the period of DHPG application or LFS delivery as indicated. **(E)** Bar histogram summarizing the averaged data within the last 10 min of the LTD recording. **P < 0.01. NS, no significance. Error bars represent SEM.

**Figure 7:**
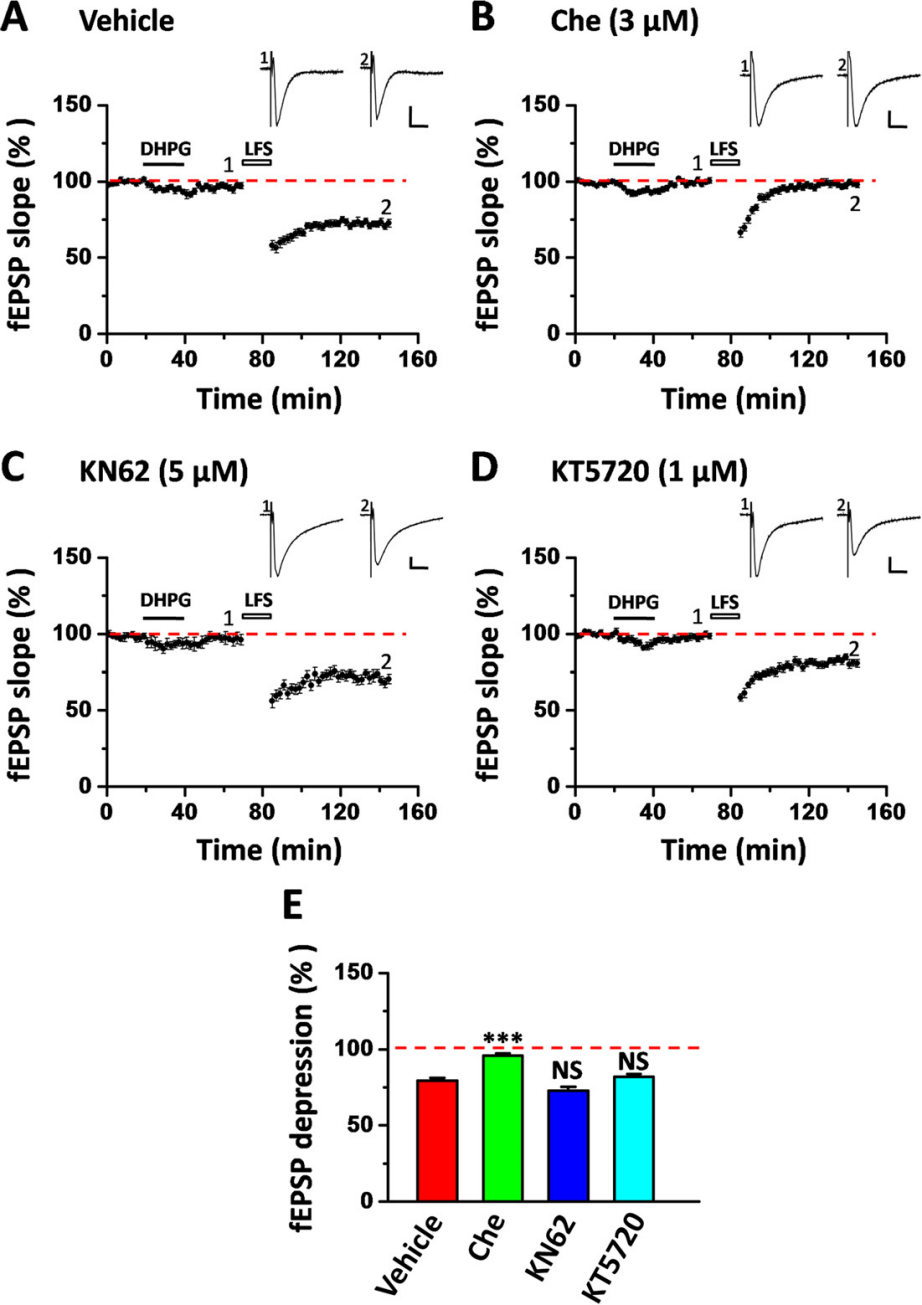
**PKC, but not CaMKII or PKA, is involved in the rescue of LFS-evoked insular LTD in the deep layer. (A)** Vehicle control group exhibited the normal rescue of insular LTD (n = 5 slices/3 mice). **(B)** Co-application of the PKC inhibitor chelerythrine (Che, 3 μM) together with DHPG (20 μM, 20 min) blocked the LTD rescue (n = 6 slices/6 mice). **(C)** CaMKII inhibitor KN62 (5 μM) could not affect the LTD recovery (n = 6 slices/5 mice). **(D)** PKA inhibitor KT5720 (1 μM) had no effect on DHPG-primed insular LTD (n = 7 slices/5 mice). Inset traces in **(A-D)** show representative fEPSPs at the time points indicated by the numbers in the graph. Calibration: 100 μV, 10 ms. Horizontal bars denote the period of DHPG application or LFS delivery as indicated. **(E)** Bar histogram summarizing the averaged data within the last 10 min of the LTD recording. ***P < 0.001. NS, no significance. Error bars represent SEM.

To probe the mechanisms underlying the metaplastic rescue of LFS-evoked LTD in the IC, we next performed pharmacological experiments using different protein kinase inhibitors, based on previous reports showing the critical roles of various protein kinases in mediating multiple forms of metaplasticity in the hippocampus [for reviews, see [61,62]. At first, we examined the involvement of PKC in the DHPG-induced priming effect, given the increasing evidence supporting the role of PKC in metaplasticity [63-65]. Co-application of a PKC inhibitor chelerythrine (Che, 3 μM) with the DHPG (20 μM, 20 min) prevented the rescue of LTD in both superficial layer (96.2 ± 2.4% of baseline within the last 10 min of recording, n = 6 slices/6 mice, *P* = 0.006, One-Way ANOVA followed by Fisher’s LSD test, Figure 6B and E) and deep layer (96.0 ± 1.4% of baseline within the last 10 min of recording, n = 6 slices/6 mice, *P* < 0.001, One-Way ANOVA followed by Fisher’s LSD test, Figure 7B and E) of the IC slice taken from tail-amputated mice. In contrast, simultaneous treatment of the IC slice with vehicle had no effect on the LTD rescue (superficial layer: 71.0 ± 2.9% of baseline, n = 5 slices/3 mice, Figure 6A and E; deep layer: 79.3 ± 1.9% of baseline, n = 5 slices/3 mice, Figure 7A and E).

Besides PKC, CaMKII and PKA have also been shown to mediate certain forms of metaplasticity [66-68]. Therefore, we also evaluated the role of these two kinases in DHPG-rescued insular LTD in the tail-amputated mice. As shown in Figure 6C-E, neither KN62 (5 μM, a CaMKII inhibitor) nor KT5720 (1 μM, a PKA inhibitor) could block the induction of LTD in the superficial layer of the IC (KN62: 65.0 ± 3.5% of baseline, n = 6 slices/5 mice, *P =* 0.595, One-Way ANOVA followed by Fisher’s LSD test; KT5720: 72.6 ± 3.9% of baseline, n = 7 slices/5 mice, *P =* 0.558, One-Way ANOVA followed by Fisher’s LSD test). Similar results were obtained in the deep layer (KN62: 72.8 ± 2.6% of baseline, n = 6 slices/5 mice, *P =* 0.382, One-Way ANOVA followed by Fisher’s LSD test; KT5720: 81.9 ± 1.9% of baseline, n = 7 slices/5 mice, *P =* 0.544, One-Way ANOVA followed by Fisher’s LSD test, Figure 7C-E). These observations are consistent with our previous results in the ACC [38], suggesting that PKC, but not CaMKII or PKA, acts as a major mediator in mGluR-evoked metaplasticity in the IC in tail-amputated animals.

## Discussion

There is considerable evidence indicating the critical role of the IC in pain perception and memory storage [2,3,16-18]. However, few studies have been conducted at the cellular level to address the synaptic basis of IC-mediated higher brain functions. Our recent work demonstrates that fast excitatory synaptic transmission in the IC is mainly mediated by postsynaptic AMPA/kainate receptors and that both LTP and LTD could be induced reliably but with different receptor mechanisms [23,44,48]. Since cortical plasticity has been proposed to be an endpoint measurement and working mechanism of chronic pain [24,69], it would be interesting to address the metaplastic effects of chronic pain experience *in vivo* on the induction of insular LTP and LTD *in vitro*. We recently report that nerve injury-induced neuropathic pain could fully occlude the subsequent induction of LTP in the IC [18]. In the present study, using a 64-channel multi-electrode array system, we further evaluated the effect of abnormal pain processing on insular LTD. We found that a two-week experience of amputation-induced peripheral injury resulted in a selective impairment of insular LTD induction by the LFS protocol, but without any effect on DHPG-induced LTD. Priming the IC slices with pharmacological activation of group I mGluRs rescued the LFS-induced LTD after amputation, which involves the activation of PKC, but not PKA or CaMKII.

### Loss of LTD in the IC after amputation

One of the central findings in this study is the loss of LFS-evoked LTD in tail-amputated IC slices. We selected two weeks after amputation as the time point for taking the IC slices for multi-channel recordings, mainly based on our previous publications showing the occurrence of marked plastic changes in the ACC at this time. Specifically, we found that peripheral amputation abolished LTD and enhanced extracellular signal-regulated kinase activation in the rodent ACC at two weeks [37-39]. Nevertheless, amputation-caused plastic changes in the brain might be time-dependent. For example, digit amputation can abolish ACC LTD and enhance hippocampal LTP at 45 min but failed to elicit any significant change in the hippocampus at 20 min or earlier [37,45]. Thus, future studies are clearly needed to investigate if tail amputation-induced loss of insular LTD is time dependent, and if so, when the metaplastic alterations are initiated and how long they can last.

The detailed mechanisms underlying this LTD abolishment are not well understood. However, our previous work revealed a similar deficit of LTD induction in the ACC from adult rats or mice subjecting to digit or tail amputation, respectively [37,38]. In addition, tissue amputation produced a rapid and prolonged enhancement of sensory responses to noxious stimulation, dramatic membrane depolarization, as well as large-scale expression of several immediate early genes and signaling molecules in the ACC [35,37,39,70], for review, see [42]. These observations allow us to speculate that enhanced postsynaptic excitability might also occur in the IC after tail amputation, which leads to the failure of LTD induction. Supporting this assertion, we found a leftward shift of input–output curves of fEPSPs in tail-amputated slices as compared to the control group. Furthermore, our recent work demonstrates that induction of insular LTD by LFS involves activation of the NMDA receptor and mGluR5 [44]. Since DHPG-induced LTD is not affected by amputation (see below), an alternative explanation for the loss of LFS-evoked LTD might be due to the changes in the expression and/ or function of NMDA receptor in the IC caused by tail amputation. Injury-induced deficits in signaling cascades at the downstream of the NMDA receptor activation may also contribute to the loss of insular LTD. Regardless of the mechanisms, loss of the ability to undergo LTD in the IC might be an essential synaptic mechanism accounting for the maladaptive central plasticity occurring after amputation [25,26,42].

### DHPG-induced LTD is not affected by tail amputation

One unexpected finding of this study is that tail amputation did not affect the induction of DHPG-LTD in superficial and deep layers of the IC. These results stand in contrast with those obtained from the adult mice ACC slices, where both electrically-induced LTD and chemically-induced LTD were significantly impaired by tail amputation [38]. The exact reasons for these discrepancies are not clear but might be due to the differences in the mGluR-targeting drugs used (DHPG vs. DHPG + MPEP) and the forebrain regions analyzed (IC vs. ACC). The conflicting observations between LFS-and DHPG-induced insular LTD could arise from their differences in the vulnerability to amputation-elicited plastic changes in the IC area. This discrepancy is also in accordance with our recent publication, demonstrating that DHPG-LTD and LFS-induced LTD represent two distinct forms of LTD co-existing in the insular synapses and do not occlude each other [44].

It is noteworthy that region-related differences might exist when considering the effects of tissue amputation on synaptic plasticity in the pain-related brain regions (summarized in Table 1). Specifically, although either tail or digit amputation triggered a complete loss of LTD in the ACC [37,38] or the IC (the present study), almost the same manipulation has no effect on LTD induction in the hippocampus or parietal cortex [37,45]. Also, partial ligation of the sciatic nerve, a well-established animal model of neuropathic pain, does not affect the induction of LFS-evoked LTD in the hippocampus [46]. These findings indicate that both ACC and IC play important roles in amputation-related cortical plasticity, and such changes are relatively selective for pain-related areas. It is unlikely due to the general stress or other non-selective factors caused by amputation. Targeting these alterations in synaptic plasticity in the brain might provide an alternative approach for the treatment of chronic pain including the phantom pain [24,25,42,69].

### mGluR-dependent rescue of insular LTD after tail amputation

It is now well-known that mGluRs activation is not only directly involved in the induction of LTP or LTD, but is also engaged in a process called metaplasticity, by which prior neuronal activity or mGluRs activation can affect the subsequent ability to exhibit synaptic plasticity [for reviews, see [61,71-73]. Nevertheless, the current literature mainly indicates the metaplastic role of mGluRs in facilitation of hippocampal LTP induction, with less emphasis placed upon their effect on LTD in cortical areas [59,60,74,75]. There are only a few reports showing that priming stimulation of group II mGluRs inhibits or facilitates the subsequent induction of LTD in CA1 or dentate gyrus, respectively [76,77], while prior activation of group I mGluRs has no effect [77]. Our recent work in the ACC revealed a facilitatory role of prior mGluR1 activation on cingulate LTD induction in the tail-amputated mice [38]. Consistently, the present study demonstrated a similar rescue of amputation-impaired insular LTD by priming treatment with DHPG (20 μM, 20 min). This is the first demonstration of the metaplasticity phenomenon in the adult mouse IC. More importantly, these observations highlight the potential of developing mGluR agonists as a novel therapeutic strategy against phantom pain (see below).

### Intracellular protein kinases mediating the pharmacological rescue

In the present study, we also examined the mechanisms of group I mGluR-mediated metaplastic rescue of insular LTD. Previously, evidence has been obtained to support the role of PKC in various types of metaplasticity, including NMDA receptor- or prior synaptic activity-induced subsequent LTP inhibition and LTD facilitation [63,78], mGluRs-mediated LTP enhancement [64,74,79] and inhibition of chemically- or electrically-induced LTD initiation [65,80]. Here, we have added the new findings that PKC activation is also an important contributing factor governing group I mGluR-mediated metaplastic rescue of amputation-impaired insular LTD. By contrast, we did not find any participation of PKA or CaMKII in the process, although there are some previous reports indicating their involvement in the regulation of metaplasticity [66-68]. Consistently, our previous work found that amputation-induced loss of LTD in the ACC was rescued by mGluR1-related PKC-dependent mechanisms [38], suggesting the important roles of PKC in mGluR-evoked metaplasticity in both ACC and IC. It is well known that group I mGluR activation can lead to intracellular calcium rise and subsequent PKC activation [81,82]. Also, the function of the NMDA receptor can be regulated through PKC-mediated signaling pathways [74,83,84]. Recently, we reported that the NMDA receptor is involved in the induction of LFS-evoked LTD in the IC [44]. It is thus reasonable to speculate that bath application of DHPG might result in significant PKC activation, which then contributes to the restoration of insular LTD through possible NMDA receptor-related mechanisms in the IC slices from tail-amputated mice. Importantly, inhibition of PKC did not affect the LTD induction in naïve IC slices [44], implying that mechanistic differences do exist between synaptic plasticity and metaplasticity in the IC.

### Clinical implications

Phantom pain is a common form of chronic pain syndrome characterized by the feeling of pain in the missing limb following amputation or deafferentation [25-27]. Until now, the clinical treatment for phantom pain is still limited and inefficient. Maladaptive plastic changes along the neuroaxis have been proposed to be associated with the occurrence and intensity of phantom pain [25,31,32,85]. Therefore, reversing these plastic changes may offer a novel way to improve the treatment of phantom pain or amputation-related brain dysfunctions. Our previous and present results reveal a loss of LFS-induced LTD in the ACC [38] and IC (the present study) following tail amputation in the adult mice, providing an alternative mechanism by which peripheral injury elicits long-lasting alterations in synaptic transmission and function in the central nervous system [37,42,47], also see Table 1]. Furthermore, we demonstrate that priming treatment with DHPG application could rescue the lost LTD in both ACC and IC after amputation, indicating that drugs acting at group I mGluRs might hold promise for the rational treatment of phantom pain by reversing amputation-evoked synaptic dysfunctions in the neocortex. From a clinical perspective, the multi-synaptic model established in the present study might be useful for further elucidating synaptic mechanisms of phantom pain in the brain, as well as screening and developing potential new drugs for treating this intractable disease in the human amputees.

## Methods

### Animals

Experiments were performed with adult (7-10 week old) male C57/BL6 mice purchased from Charles River (Quebec, Canada). All animals were fed in groups of three per cage under standard laboratory conditions (12 h light/12 h dark, temperature 22-26°C, air humidity 55-60%) with *ad libitum* water and mice chow. The experimental procedures were approved by the Institutional Animal Care and Use Committee of The University of Toronto. All animals were maintained and cared for in compliance with the guidelines set forth by the International Association for the Study of Pain [86]. The number of animals used and their suffering were greatly minimized.

### Drugs

The drugs used in this study include: DHPG, chelerythrine, KT5720 and KN62. Among them, chelerythrine and DHPG were dissolved in distilled water, while KT5720 and KN62 were prepared in dimethyl sulfoxide (DMSO) as stock solutions for frozen aliquots at -20°C. All these drugs were diluted from the stock solutions to the final desired concentration in the artificial cerebrospinal fluid (ACSF) before immediate use. The diluted DMSO in ACSF had no effect on baseline synaptic transmission and plasticity. Chelerythrine, KT5720 and KN62 were purchased from Tocris Cookson (Bristol, UK) and DHPG was obtained from Abcam Biochemicals (Cambridge, UK). The doses for each compound were chosen based on our preliminary experiments and on relevant information from previous papers [38,44,77]. For the pharmacological rescue of insular LTD, DHPG (20 μM) with or without the drugs was bath applied for 20 min and then washed out for 30 min prior to LTD induction.

### Tail amputation

The major procedures for tail amputation are in accordance with those described previously [38,45,87]. After anesthesia with gaseous isoflurane, the mouse was gently put in a box where a 2.5 cm length of the tail tip was removed using surgical scissors. A drop of Krazy Glue was used to stop bleeding. The mice typically recovered from anesthesia within 3-5 min. Amputated animals did not exhibit any neurological deficits or abnormal behaviors when returned to the home cage. For the sham control group, mice were anesthetized for the same period of time without any surgery. Procedure was executed with caution to minimize handling-induced stress in the mice. In the present study, we performed electrophysiological recordings at 2 weeks after tail amputation (Figure 1A), on the basis of our previous reports showing an evident plastic change in the ACC at this time point [37-39].

### Insular slice preparation

The general procedures for making IC slices are similar to those described previously [18,23,44,48]. Briefly, mice were anesthetized with a brief exposure to gaseous isoflurane and decapitated. The entire brain was rapidly removed and immersed into a cold bath of oxygenated (95% O_2_ and 5% CO_2_) ACSF containing (in mM): NaCl 124, KCl 2.5, NaH_2_PO_4_ 1.0, MgSO_4_ 1, CaCl_2_ 2, NaHCO_3_ 25 and glucose 10, pH 7.35-7.45. After cooling for 1-2 min, appropriate portions of the brain were then trimmed and the remaining brain block was glued onto the ice-cold stage of a vibrating tissue slicer (Leika, VT1000S). Following this, three coronal IC slices (300 μm) were obtained at the level of corpus callosum connection and transferred to an incubation chamber continuously perfused with oxygenated ACSF at 26°C. Slices were allowed to recover for at least 2 h before any electrophysiological recording was started.

### Multi-channel field potential recordings

A commercial 64-channel multi-electrode array system (MED64, Panasonic Alpha-Med Sciences, Japan) was used for extracellular field potential recordings in this study. Procedures for preparation of the MED64 probe and multi-channel field potential recordings were similar to those described previously [23,38,43,44]. The device had an array of 64 planar microelectrodes, each 50 × 50 μm in size, arranged in an 8 × 8 pattern (inter-electrode distance: 150 μm). Before use, the surface of the MED64 probe was treated with 0.1% polyethyleneimine (Sigma, St. Louis, MO, USA) in 25 mM borate buffer (pH 8.4) overnight at room temperature. After incubation, one slice was positioned on the MED64 probe in such a way that the IC area was entirely covered by the recording dish mounted on the stage of an inverted microscope (CKX41, Olympus). The relative location of the IC slice with the probe followed the anatomical atlas [88], also see Figure 1B]. Once the slice was settled, a fine mesh anchor (Warner Instruments, Harvard) was carefully disposed to ensure slice stabilization during recording. The slice was continuously perfused with oxygenated, fresh ACSF at the rate of 2-3 ml/min with the aid of a peristaltic pump (Minipuls 3, Gilson) throughout the entire experimental period.

After a 15-20 min recovery of the slice, one of the 64 available planar microelectrodes was selected from the 64-switch box for stimulation by visual observation through a charge-coupled device camera (DP70, Olympus) connected to the inverted microscope. For test stimulation, monopolar, biphasic constant current pulses (0.2 ms in duration) generated by the data acquisition software (Mobius, Panasonic Alpha-Med Sciences) were applied to the deep layer (layer V-VI) of the IC slice at 0.008 Hz (red dot in Figure 1B). The fEPSPs evoked at both the deep layer and the superficial layer (layer I-III) of the IC slice were amplified by a 64-channel amplifier, displayed on the monitor screen and stored on the hard disk of a microcomputer for offline analysis. After selecting the best stimulation site and stabilizing the baseline synaptic responses, an input–output curve was first determined for each group using the measurements of fEPSP slope or the number of activated channels (output) in response to a series of ascending stimulation intensities from 6 μA to 24 μA by every 2 μA step (input). For the LTD induction, the intensity of the test stimulus was adjusted to elicit 40-60% of the maximum according to the input–output curves. Stable baseline responses were then monitored for at least 20 min before delivering a classical LFS protocol (1 Hz, 900 pulses, with the same intensity as baseline recording) to induce NMDA receptor-dependent LTD. In another set of experiments, DHPG (100 μM, 20 min) was bath applied to induce another form of LTD [44]. After LFS or DHPG application, the test stimulus was repeatedly delivered once every 2 min for 1 h or 50 min to monitor the time course of insular LTD.

### Data analysis

All multi-channel electrophysiological data were analyzed offline by the MED64 Mobius software. For quantification of the input–output relationship, the slope of fEPSP was measured and expressed as the percentage of 8 μA value according to different layers (superficial layer and deep layer). It is notable that data from the stimulation intensity of 6 μA were not included in the slope analysis due to the much fewer fEPSP evoked at this low intensity. The number of activated channels evoked at different stimulation intensities was also counted in a blind manner. For quantification of the LTD data, the initial slope of fEPSPs was measured by taking the rising phase between 10% and 90% of the peak response, normalized and presented separately in both superficial and deep layers as a percentage change from the baseline level. The degree of LTD in each experiment was shown as the value obtained at 50 min or 60 min after DHPG or LFS treatment, respectively. For evaluation of the drug effects on rescued LTD, the averaged value of the last 10 min of the recording was compared statistically. Furthermore, the number of activated channels (over 20% of baseline, i.e. the amplitude goes over -20 μV) vs. the LTD-showing (depressed by at least 15% of baseline) channels was counted and expressed as the induction ratio of LTD (number of LTD-occurring channels /number of all activated channels × 100%). All data are shown as mean ± S.E.M. When necessary, the statistical significance was assessed by two-tailed Student’s t test and one-way ANOVA (followed by post doc Fisher’s LSD test) using the Sigma Plot software. *P* < 0.05 was assumed as statistically significant.

## Abbreviations

ACC: Anterior cingulate cortex; ACSF: Artificial cerebrospinal fluid; CaMKII: Calcium/calmodulin-dependent protein kinase II; DHPG: (RS)-3,5-dihydroxyphenylglycine; DMSO: Dimethyl sulfoxide; fEPSP: Field excitatory postsynaptic potential; IC: Insular cortex; LFS: Low-frequency stimulation; LTD: Long-term depression; LTP: Long-term potentiation; MED64: 64-channel multi-electrode dish; mGluR: Metabotropic glutamate receptor; PKA: Protein kinase A; PKC: Protein kinase C.

## Competing interests

The authors declare that they have no competing interests.

## Authors’ contributions

M.-G.L. performed the experiments, analyzed data and drafted the manuscript; M.Z. conceived and designed the research and finished the final version of the manuscript. Both authors read and approved the final manuscript.
